# HIV-1 drug resistance in recently HIV-infected pregnant mother’s naïve to antiretroviral therapy in Dodoma urban, Tanzania

**DOI:** 10.1186/1471-2334-13-439

**Published:** 2013-09-21

**Authors:** Francesco Vairo, Emanuele Nicastri, Giuseppina Liuzzi, Zainab Chaula, Boniface Nguhuni, Nazario Bevilacqua, Federica Forbici, Alessandra Amendola, Lavinia Fabeni, Pasquale De Nardo, Carlo Federico Perno, Angela Cannas, Calistus Sakhoo, Maria Rosaria Capobianchi, Giuseppe Ippolito

**Affiliations:** 1 “L. Spallanzani” National Institute for Infectious Diseases- INMI, Via Portuense 292, 00149 Rome, Italy; 2 Resource Centre for Infectious Diseases, Dodoma Regional Hospital, Dodoma, Tanzania

**Keywords:** HIV-drug resistance, MTCT, HIV-genotype, Low-resources countries

## Abstract

**Background:**

HIV resistance affects virological response to therapy and efficacy of prophylaxis in mother-to-child-transmission. The study aims to assess the prevalence of HIV primary resistance in pregnant women naïve to antiretrovirals.

**Methods:**

Cross sectional baseline analysis of a cohort of HIV + pregnant women (HPW) enrolled in the study entitled Antiretroviral Management of Antenatal and Natal HIV Infection (AMANI, peace in Kiswahili language). The AMANI study began in May 2010 in Dodoma, Tanzania. In this observational cohort, antiretroviral treatment was provided to all women from the 28^th^ week of gestation until the end of the breastfeeding period. Baseline CD4 cell count, viral load and HIV drug-resistance genotype were collected.

**Results:**

Drug-resistance analysis was performed on 97 naïve infected-mothers. The prevalence of all primary drug resistance and primary non-nucleoside reverse-transcriptase inhibitors resistance was 11.9% and 7.5%, respectively. K103S was found in two women with no M184V detection. HIV-1 subtype A was the most commonly identified, with a high prevalence of subtype A1, followed by C, D, C/D recombinant, A/C recombinant and A/D recombinant. HIV drug- resistance mutations were detected in A1 and C subtypes.

**Conclusion:**

Our study reports an 11.9% prevalence rate of primary drug resistance in naïve HIV-infected pregnant women from a remote area of Tanzania. Considering that the non-nucleoside reverse-transcriptase inhibitors are part of the first-line antiretroviral regimen in Tanzania and all of Africa, resistance surveys should be prioritized in settings where antiretroviral therapy programs are scaled up.

## Background

In 2010, 5 million people had access to HIV treatment in Sub-Saharan Africa, about 49% of those in need. In Eastern and Southern Africa, 56% of eligible patients had access to therapy, versus 10% treated in 2009 [1]. The scale-up of HIV treatment in low- and middle-income countries has been crucial to substantially reduce AIDS-related morbidity and mortality as well as mother-to-child-transmission (MTCT). As access to antiretroviral therapy expands, HIV drug resistance (HIVDR) inevitably emerges because of HIV’s high mutation rate, viral recombination, and the patient’s need for sustained, lifelong treatment. The probability of drug resistance escalation during treatment has been estimated at 27% every 6 years [2]. The HIVDR insurgence may be related to a different mechanism. It could be due to drug pressure in patients receiving antiretroviral therapy (ART) because of suboptimal adherence, pharmacodynamic factors, or use of inadequate or suboptimal regimes. In recently infected individuals, HIVDR may be transmitted from one individual to another. Finally, HIVDR can be transmitted or acquired in individuals with chronic infections.

The implications of transmitted drug resistance are a cause of concern for the scaling up of HIV programs, as HIV resistance also affects the efficacy of MTCT prophylaxis.

Drug sensitivity testing (DST) is the standard of care in industrialized countries, but is rarely available in resource-limited settings due to high costs and stringent requirements for storage and transport of plasma. With the introduction of antiretroviral drugs in low-resource countries (known for the largest assortment of non-B subtypes), gaining a better understanding of the responsiveness to antiretroviral therapy and HIV-1 drug resistance in non-B strains has become a priority. In such settings, patients who do not respond to therapy are often blindly switched from a non-nucleoside reverse transcriptase inhibitor (NNRTI) to a protease inhibitor (PI)-based regimen. However, since treatment failure is detected late in most patients (at a stage when widespread resistance is common), the risk of switching to regimens with limited efficacy increases.

Among determinant factors driving the emergence of HIVDR, virus- related factors play a crucial role in the susceptibility to drug- resistance mutations. Viral subtypes other than B share different ARV susceptibilities compared to HIV-1 non-B subtypes, which are naturally more or less susceptible to specific drugs. Different results were obtained in several studies that have compared the prevalence of drug-related mutations in different HIV-1 non-B subtypes [3]: the recombinant form CRF02_AG is reported to be more susceptible to nelfinavir (NFV) and ritonavir (RTV) than C and F subtypes; G subtype is more sensitive to tipranavir (TPV) and lopinavir (LPV) than other subtypes [4], and C subtype has a greater risk of developing resistance to tenofovir (TDF) [5]. In a Ugandan study [6], the K103N mutation was relatively more frequent in C subtype- infected women failing NNRTI-based therapy than in both A and D subtypes. The G190A/S mutation was considered a common polymorphism in Israeli C subtype patients, but not in Indian C subtype individuals [7,8]. Despite the variability of non-B HIV-1 subtypes in viral mutational patterns and *in vitro* susceptibility, the benefit of treatment programs clearly outweigh the risks of emerging HIV DR [3-8].

Future clinical studies designed to provide clinical and virological data in non-B strains are of great interest. Additional information on the prevalence of drug-resistance mutations in naïve HIV populations could be crucial for tailoring combination regimens. Furthermore, it could help clinicians to decide whether DST prescription is necessary before initiating therapy.

This study aims to assess the prevalence of HIV drug-related resistance and the circulation of non-B subtype in pregnant women naïve to antiretrovirals in Dodoma region, central mainland Tanzania.

## Methods

### Study design

The data provided are part of a nested case-control study of HIV resistance outcome among the HIV + pregnant women (HPW) enrolled in the study entitled Antiretroviral Management of Antenatal and Natal HIV Infection (AMANI, peace in Kiswahili language). The AMANI study is an interventional study which aims to assess the feasibility of ART use for preventing MTCT in a cohort of HIV-infected pregnant women. HAART is provided to all women starting at the 28^th^ week of gestation until the end of the breastfeeding period, within an integrated MTCT prevention program. A systematic screening during a formal interview on previous ART use including single-dose NVP is performed. Baseline CD4 cell count, viral load, and HIV drug- resistance genotypes are collected at baseline, during pregnancy and lactation.

The current study analyzed a subgroup of 97 pregnant women naïve to any antiretroviral treatment. In order to be certain that there was no previous exposure to any ARV, women were included in the study only if the first HIV positivity was discovered during the current pregnancy. The AMANI study was approved by the Italian Ethical Board of the “L. Spallanzani” National Institute for Infectious Diseases in November 2009 and by the Tanzanian Medical Research Coordinating Committee of the National Institute of Medical Research (NIMR), with certificate no. NIMR/HQ/R.8a/Vol.IX/907 in December 2009. All recruited women provided written informed consent.

### HIV sequencing

HIV genotype analysis was performed on plasma samples by using a commercially available HIV genotyping kit (ViroSeq HIV-1 Genotyping System version 2.0, Abbott Molecular).

In brief, RNA was extracted using a commercially available kit (QIAamp RNA Viral Mini kit, Qiagen), retrotranscribed by murine leukaemia virus RT, and amplified with ampliTaq Gold polymerase enzyme. Pol amplified products (containing the entire Protease (99-aa) and the first 320 amino acids of the Reverse Transcriptase) were full-length sequenced in sense and antisense orientations, using seven different overlapping sequence-specific primers by an automated sequencer (ABI 3130, Applied Biosystems, Foster City, CA, USA) [9,10]. Sequence data were analyzed by a specific HIV genotyping system software that automatically assembles the seven sequence segments into a consensus sequence, which is then compared to a B reference strain. Sequences having a mixture of wild-type and mutant residues at single positions were considered to have a mutation at that position. When the mixture was between two different mutations, both mutations were considered and reported. To classify and identify polymorphisms and mutations associated with resistance to ARVs, the FASTA sequences of the PR and RT were analyzed using the freely available SDRM-2009 algorithm available in the Calibrated Population Resistance tool (CPR), version 6.0 beta (http://cpr.stanford.edu/cpr.cgi). The SDRM algorithm (the SDRM worksheet shows all of the mutations present on the ANRS, HIVdb, IAS-USA, Los Alamos, and Rega algorithm lists) [11] was applied to determine the prevalence of primary ARV- resistance mutations, using a list of drug-resistance mutations that provide an estimate of resistance transmission according to the WHO guidelines (http://hivdb.stanford.edu/cgi-bin/AgMutPrev.cgi).

### Genotypic sub typing

Pol subtype was determined using phylogenetic analysis on HIV-1 pol-sequences. Briefly, the sequences were aligned with HIV-1 reference sequences of all subtypes (http://www.hiv.lanl.gov). The alignment was edited using the BioEdit program version 7.0.5.3. The phylogenetic analysis of *pol* aligned sequences was performed by the maximum-likelihood method of MEGA version 5.05. The transversion model (GTR + I + G) of nucleotide substitution was chosen using the Kimura two-parameter model as the best-fitting evolution model for tree reconstruction. The reliability of the branching patterns was evaluated by bootstrapping (1,000 replicates). Only bootstrap values >70% were evaluated. Subtype classification was also confirmed by REGA subtype tool (http://www.bioafrica.net/rega-genotype/html/subtypinghiv.html) and COMET subtype tool (http://comet.retrovirology.lu/). To improve the accuracy of subtype recombinant forms and unique forms, RDP3 software (http://web.cbio.uct.ac.za/~darren/rdp.html) and Splits Tree software (http://www.splitstree.org/) were used.

### Statistical analysis

Descriptive statistics were used to analyze the epidemiological data. Chi- square tests were used to assess differences between groups with reference to the occurrence of drug-resistance mutations. A univariate analysis was performed to examine possible demographic, clinical and viro-immunological factors related to the occurrence of drug-resistance mutations. The Chi-square test was used for the categorical variable. The T-test was used for comparison of the means for the quantitative variable; Wilcoxon signed rank was used for comparison of medians. The significant level was set at 0.05. All the analyses were performed using SPSS for Windows 12.0 (SPSS Inc, Chicago, Illinois 60606, USA).

## Results

**Figure 1:**
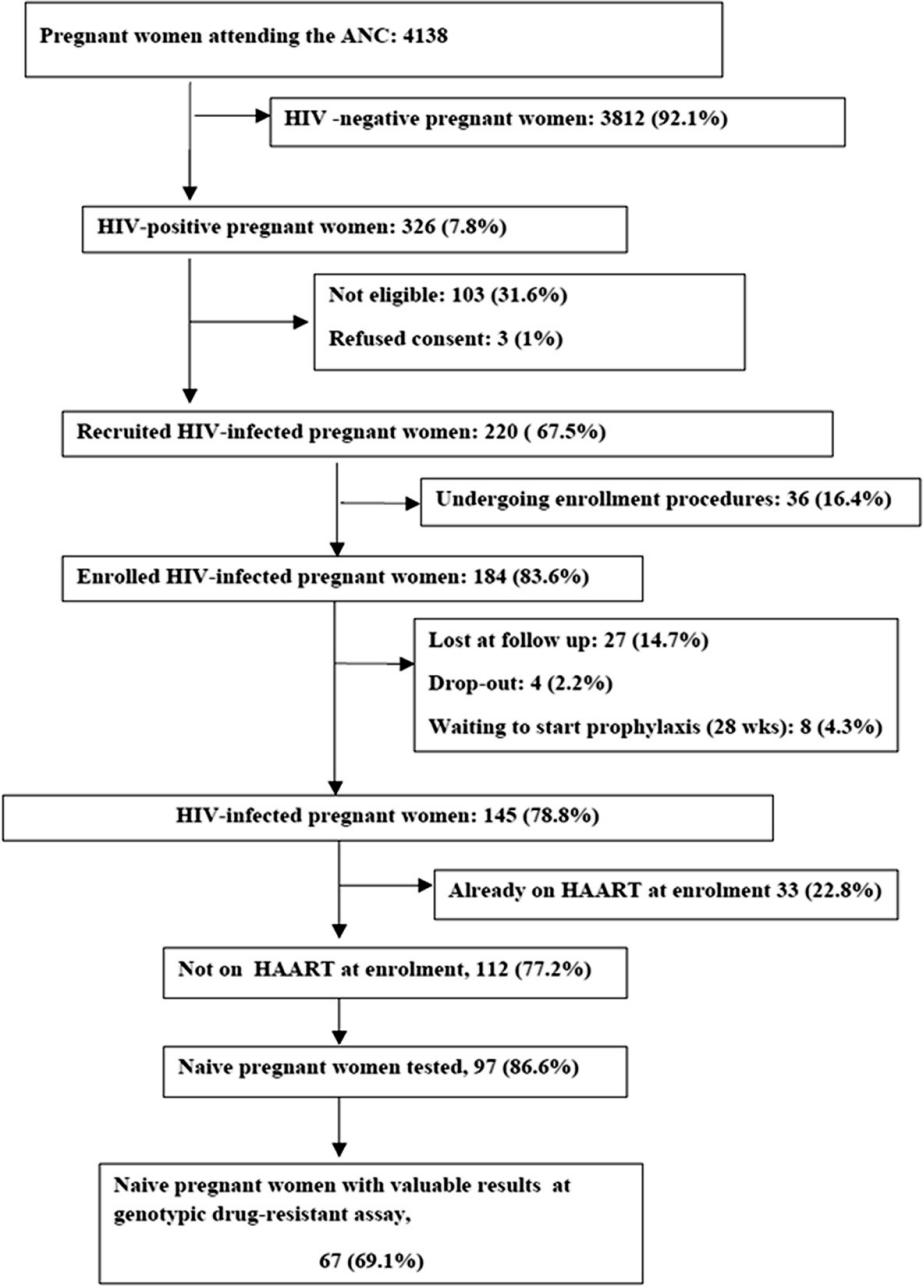
Total clinic attendance and patients recruited in the AMANI Study in Makole Health Centre after 19 months: enrollment algorithm.

The enrollment phase of the AMANI study started on May 2010 at the Makole Urban Health Centre (Makole UHC) in the municipality of Dodoma (Figure 1). During the nineteen months, 4,138 pregnant women attended the Ante Natal Clinic (ANC), and 326 (7.8%) of these were found to be affected by HIV infection. Among them, 103 (31.6%) HPW were not eligible for the study (i.e. attending the ANC with gestational age >28 weeks), 3 (1%) refused to participate (due to fear of drug side effects), and 220 (67.5%) were included in the study. Thirty-six of them (16.4%) are currently under evaluation for inclusion in the study. Twenty-seven (14.7%) of the remaining 184 (83.6% of 220) did not return after the enrollment visit and 12 (6,5%) dropped out. Of the remaining 145 (78.8%) patients, 33 (22.8%) were already on HAART at enrollment. Among the 112 (77.2%) HPWs not on HAART at baseline, 85 (75.9%) started ART for prevention of MTCT at the 28^th^ week of gestation, and 27 (24.1%) were eligible for therapy and started HAART (Figure 1).

Of the 112 patients originally not on HAART, 97 mothers with a first HIV positivity during the current pregnancy and with no reported previous ART use were tested for drug resistance before starting any antiretroviral drug (Table 1): 53 (54.6%) and 27 (27.8%) were defined as WHO stage 1 and 2, respectively. Unprotected heterosexual intercourse was the risk factor for HIV infection reported by all HPWs in the study. At baseline, the median CD4 count was 392 (IQR, 260-528) cells/mm3 and Median log_10_ HIV RNA copies/mL was 4.80 (IQR, 4.03-9.28). The HIV genotypic drug-resistance assay provided valuable results in 67 cases (69.0%). The overall prevalence of primary drug resistance was 11.9% (8/67; 95% CI 0.04-0.20); the prevalence of primary drug class-specific resistance was 1.5% (1/67; 0.01-0.04) for nucleoside reverse-transcriptase inhibitors (NRTIs) and 7.5% (5/67; 0.01-0.14) for NNRTIs. K103S and M41L were found in two and one women, respectively, while M184V was not detected. Interestingly, L89M protease polymorphism (potentially associated with resistance to fosamprenavir, and to a lesser extent to darunavir and lopinavir) was commonly detected with a 76.1% prevalence, equally distributed in all different non-B clades.

**Table 1:** Baseline clinical and viro-immunological characteristics of 97 HIV-infected pregnant women naive for any antiretroviral drug

**Characteristics**	**All pregnant women**
	**n° 97**
Age, years, mean ± SD; median, (IQR)	29,±5; 28 (25–32)
Gestational age at enrolment, week, mean ± SD	20 ± 5
Height at screening, cm, median (IQR)	155 (153–159)
Race/ethnicity, n. (%)	
Black African	97 (100)
Education, n. (%)	
Primary school	66 (68.0)
Secondary school	17 (17.5)
Other or unknown	14 (14.5)
Number of pregnancies, mean ± SD	3 ±1
Number of deliveries, mean ± SD	2 ±1
Number of children, mean +/-SD	1 ±1
HIV risk factor, n. (%)	
Unprotected heterosexual relationship	97 (100)
WHO stage, n. (%)	
1	53 (54.6)
2	27 (27.8)
3	15 (15.5)
4	2 (2.1)
Days since HIV diagnosis, median (IQR)	136 (94–164)
MedianCD4+ cells at enrolment /μL (IQR)	392 (260–528)
Median log_10_ HIV RNA copies/mL (IQR)	4.80 (4.03–9.28)
Eligible for HAART, n (%)	19 (19.6%)
Positive VDRL assay, n (%)	3 (3.1)

Note. *IQR* Interquartile Range, *SD* Standard Deviation.

K103N and M41L mutations were on five lists and the K103S mutation was on four lists.

**Figure 2:**
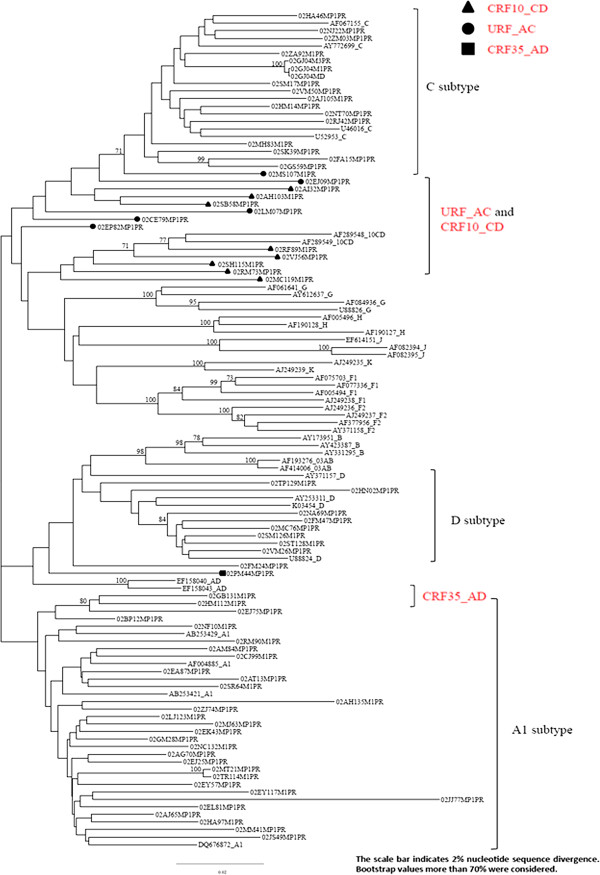
Phylogenetic tree of different non-B subtypes in 67 HIV-infected naive pregnant women with valuable results from the HIV genotypic drug-resistance assay.

The HIV-1 A subtype was the most commonly identified (36/67, 53.7%) with a high prevalence of A1 subtype (31/67, 47%), followed by C (14/67, 21%), and D (9/67, 13%). Furthermore, some circulating recombinant forms (CRFs): CRF10_CD (8/67, 12%), CRF35_AD recombinant (1/67, 1%) and some unique recombinant forms (URFs) A1/C recombinant (4/67, 6%) were reported. The phylogenetic tree appears to show a cluster of related infections in this geographical area, particularly in A1 and C subtypes (Figure 2). Finally, two K103S drug-related mutations and one K103N polymorphism were all clustered in the A1 subtype. Pregnant women with HIV viral strains harboring drug-resistance mutations before any antiretroviral treatment did not significantly differ from women with wild-type HIV in terms of demographic, clinical, virological and immunological parameters (Table 2).

**Table 2:** Baseline clinical and viro-immunological characteristics of 67 HIV-infected naive pregnant women with valuable results from the HIV genotypic drug-resistance assay

**Characteristics**	**HIV-infected pregnant women with a valuable result at HIV genotype n° 67**		**p value**
	**Women with wild-type genotype n° 59**	**Women with any drug-resistant mutation n°8**	
Age, years, mean ± SD	28.6 ± 5.3	30.6 ± 4.7	0.3
Gestational age at enrolment, week, mean ± SD;	20.8 ± 4.9	17.2 ± 4.4	0.06
Height at screening, cm, mean ± SD	155.9 ± 5.9	155.3 ± 3.2	0.8
Race/ethnicity,			
Black African, n. (%)	59 (100)	8 (100)	1
Education, n. (%)			0.3
Primary school	36 (61)	7 (87.5)	
Secondary school	10 (16.9)	1 (12.5)	
Other or unknown	13 (22)	0 (0)	
Number of pregnancies, mean ± SD	2.9 ±1.5	2.6 ±1.8	0.6
Number of deliveries, mean ± SD	1.8 ±1.5	1.6 ±1.8	0.7
Number of children, mean ± SD	1.4 ±1.2	1.6 ±1.8	0.6
HIV risk factor, n° (%)			
Unprotected heterosexual relationship	59 (100)	8 (100)	1
WHO stage, n° (%)			0.9
1	35 (59)	4 (50)	
2	15 (25)	3 (37.5)	
3	8 (14)	1 (12.5)	
4	1 (2)	0	
MedianCD4+ cells at enrolment /μL (IQR)	406 (297-530)	396 (240-461)	0.7
Days since HIV diagnosis, mean ± SD	102 ±182	117 ±28	0.8
Median log_10_ HIV RNA copies/mL (IQR)	9.28 (4.82-10.71)	7.94 (3.58-9.49)	0.3
Eligible for HAART, n (%)	9 (15.5)	2 (25)	0.5
Positive VDRL assay, n (%)	1 (1.9)	1 (12.5)	0.1

Note. Demographic, clinical and viro-immunological data were analyzed with Chi- square test and univariate analysis (significant level was set at 0.05).

## Discussion

This cross sectional study shows an 11.9% overall prevalence of HIV primary drug resistance with a 7.5% NNRTI-related resistance in naïve pregnant women.

The recent HIV drug resistance report, released by WHO in November 2012 [12], reports the available data on the estimated prevalence of HIVTDR between 2003 and 2010 in 72 surveyed areas. WHO recommends a minimum-resource method to assess HIVTDR in resource-limited countries where transmitted HIV drug resistance is likely to be seen first (such as in urban areas). Of the 72 surveys, 52 (72.2%) had a low prevalence of resistance to all drug classes and 20 (27.8%) had a moderate prevalence classification of resistance to ≥1 antiretroviral drug class). The first reports from Tanzania in 2005 showed a 4% and 9% NNRTI resistance in naïve populations from the Kagera-Kilimanjaro regions and Dar-es-Salaam urban area, respectively [13,14]. More recently, in 2011, a 14.7% HIVDR prevalence in a naïve population was reported in Mwanza [15]. Authors combined drug-resistance prevalence data obtained from both peripheral blood mononuclear cells (PBMC) and plasma, whereas only 4 samples (9.5%) resulted positive at bulk sequencing assay from plasma [15]. The NNRTI prevalence rates observed in these surveys are slightly higher than those observed in previous reports in other eastern and western African countries.

Reports showed the effect of a proper timing for introduction of ART, as a proxy for the amount of circulating drug-resistance HIV-1 strains at the population level, and level of primary resistance [16]. The overall sample-weighted drug resistance prevalence was 5.6% (139 of 2436; 95% CI 4.6–6.7), ranging from 1.1% (two of 176; 0.0–2.7) in Pretoria, South Africa, to 12.3% (22 of 179; 7.5–17.1) in Kampala, Uganda [16].

Regarding PI mutations, no major resistance mutations were observed in our study. Nevertheless, the 76.1% prevalence of the L89M protease polymorphism raises concern. Some amino acid polymorphisms occur at sites that have been associated with drug resistance in the B-subtype virus [9]. The L89M mutation increases the catalytic efficiency and vitality of the HIV-1 protease gene in the presence of other protease mutations in non-B African viral subtypes [17] and can determine a low accumulation of primary protease mutations in non-B subtypes [18,19]. These findings suggest that in addition to the primary drug-related mutations already described in B clades, particular attention should be paid to some natural polymorphisms in the therapeutic management of patients infected by HIV-1 non-B subtypes.Our study aimed to determine the prevalence of transmitted HIV drug-resistance mutations among untreated patients and also provided novel data on the HIV-1 variants that circulate in Tanzania. We confirmed previous results that reported a high genetic diversity in the number of co-circulating variants with the predominance of A clade (53.7%), and a high prevalence of the A1 subtype (47%). Different from a 2004 report that described a low detection of drug resistance in A subtype compared to D subtype [20], we reported that the primary NNRTI drug-related mutations were all clustered in the A1 subtype. This variant was described as one of the most prevalent variants among young adults in Tanzania, Dar-es-Salaam [14] and adults in the Kilimangiaro, Kagera and Mwanza regions [13,15]. Thus, the implementation of a surveillance study on the molecular epidemiology of different HIV strains appears strictly complementary to the data obtained from prevalence studies of drug-related mutations using bulk sequencing.

Several limitations were encountered in our study. Despite systematic screening of previous ART use, the mean number of pregnancies is three and the risk of unreported ART use in the AMANI cohort may be significant. Nevertheless, most HIVDR mutations were reported in parous women; the AMANI study was conducted in a single area; a single population was targeted, and the estimates of drug resistance have wide CIs. However, despite all these considerations, the 11.9% prevalence of drug resistance in a naïve population where the first-line antiretroviral regimen is still based on a NNRTI-based HAART raises concern. Further work should be done to determine if resistance is a consequence of short-term exposure during pregnancy or if in fact these individuals had already accessed ARVs. Together, this information could be used to guide the development of ART policy guidelines in Tanzania. Against this background, the increasing rates of antiretroviral resistance in adults and children from low-income settings represent a potential threat and urgent actions are needed. First, human and resource efforts should be doubled to deploy proven effective preventive methods. Second, early and sustained ART use for preventing MTCT must be fully embraced [21] and the recent 2012 WHO programmatic update on HIV PMTC transmission [22] should be strongly supported to provide the option B-plus, the use of a life-long triple therapy for the pregnant women.

## Conclusion

Despite all the discussed considerations, the 11.9% prevalence of drug resistance in a naïve population where the first-line antiretroviral regimen is still based on a NNRTI-based HAART raises concern. Further work should be done to determine if resistance is a consequence of short-term exposure during pregnancy or if in fact these individuals had already accessed ARVs. Together, this information could be used to guide the development of ART policy guidelines in Tanzania. Against this background, the increasing rates of antiretroviral resistance in adults and children from low-income settings represent a potential threat and urgent actions are needed. First, human and resource efforts should be doubled to deploy proven effective preventive methods. Second, early and sustained ART use for preventing MTCT must be fully embraced [21] and the recent 2012 WHO programmatic update on HIV PMTC transmission [22] should be strongly supported to provide the option B-plus, the use of a life-long triple therapy for the pregnant women.

## Competing interests

The authors declare that they have no conflict of interest.

## Authors’ contributions

FV, EN, NB, GL and GI designed and supervised the study. BN and ZC carried out the patient enrollment. CS and AC supervised the laboratory diagnostic analysis at DRH, Tanzania. FF, AA, LF, CFP and MRC performed the HIV-Genotype analysis at INMI. PDN, EN, FV and AC analyzed data and contributed to the preparation of the manuscript. All authors read and approved the final manuscript.

## Pre-publication history

The pre-publication history for this paper can be accessed here:

http://www.biomedcentral.com/1471-2334/13/439/prepub
